# Self-Presentation Strategies, Fear of Success and Anticipation of Future Success among University and High School Students

**DOI:** 10.3389/fpsyg.2017.01884

**Published:** 2017-10-27

**Authors:** Natasza Kosakowska-Berezecka, Paweł Jurek, Tomasz Besta, Sylwia Badowska

**Affiliations:** ^1^Institute of Psychology, University of Gdansk, Gdansk, Poland; ^2^Faculty of Management, University of Gdansk, Gdansk, Poland

**Keywords:** gender pay gap, anticipation of success, self-stereotyping, self-promotion, backlash avoidance model

## Abstract

The backlash avoidance model (BAM) suggests women insufficiently self-promote because they fear backlash for behavior which is incongruent with traditional gender roles. Avoiding self-promoting behavior is also potentially related to associating success with negative consequences. In two studies we tested whether self-promotion and fear of success will be predictors of lower salaries and anticipation of lower chances of success in an exam. In study 1, prior to the exam they were about to take, we asked 234 students about their predictions concerning exam results and their future earnings. They also filled scales measuring their associations with success (fear of success) and tendency for self-promotion. The tested model proved that in comparison to men, women expect lower salaries in the future, anticipate lower test performance and associate success with more negative consequences. Both tendency for self-promotion and fear of success are related to anticipation of success in test performance and expectations concerning future earnings. In study 2 we repeated the procedure on a sample of younger female and male high school pupils (*N* = 100) to verify whether associating success with negative consequences and differences in self-promotion strategies are observable in a younger demographic. Our results show that girls and boys in high school do not differ with regard to fear of success, self-promotion or agency levels. Girls and boys anticipated to obtain similar results in math exam results, but girls expected to have higher results in language exams. Nevertheless, school pupils also differed regarding their future earnings but only in the short term. Fear of success and agency self-ratings were significant predictors of expectations concerning future earnings, but only among high school boys and with regard to earnings expected just after graduation.

## Introduction

Compensation statistics gathered in Europe and the United States (Blau et al., 2014; Eurostat, 2016; Kijewska, 2017) invariably suggest a significant pay gap between men and women. In the literature, two primary explanations account for this gap: the human capital theory and compensation differentials between women and men. The human capital theory suggests different skill levels and economy locations (by occupation and industry) account for over one half of the gender wage gap and existing gender discrimination in the labor market (Levanon et al., 2009; Blau and Kahn, 2016). Recent research also suggests that differences in personality traits provide a useful explanation for existing gender pay gap. Men tend to value money, have greater self-esteem, take greater risks, feel more personal control over their lives and pursue competitive situations more often than women (Blau and Kahn, 2016). On the other hand, the differences in personality traits are strongly linked to existing gender stereotypes - Bertrand et al. (2015) relate the existing gender pay gap to cultural gender stereotypes and address the effects of traditional gender roles on the financial outcomes of men and women.

Gender stereotypes link femininity with communality and interdependence and masculinity with agency and independence (Williams and Best, 1990). Since gender stereotypes typically influence male and female identity, these stereotypes also define acceptable male and female behavior. People tend to meet these norms to avoid social and economic backlash for gender incongruent behavior (Rudman and Fairchild, 2004; Akerlof and Kranton, 2010). In our article we focus on two psychological characteristics of women and men, such as willingness to self-promote and seeing one's successes as having negative consequences—these two variables potentially relate to women's expectations concerning their future earnings and chances for success in general. Both modest self-promotion and fearing that success can have negative consequences are related to prescriptive contents of female gender roles stereotypes.

Both men and women tend to estimate higher salaries for men. This phenomenon is known as the “salary estimation effect” (Williams et al., 2010). Women typically require less compensation than men, and since they demand less, they get paid less. Major et al. (1984b) demonstrated that job applicants who had lower salary expectations were paid significantly less money by their employers than equally qualified applicants who demanded higher financial compensation—hence when you demand less you receive less. It is important to note that lack of self-confidence is not the cause of women's under-compensation, rather, women tend to accept lower compensation than men (Small et al., 2007). Their lower salary demands might be also related to women's comparatively modest self-presentation skills (Moss-Racusin and Rudman, 2010)—research shows that women are ready to accept less not due to assumed lack of ability but rather out of their concern for violating gender norms which orders them to be modest and not to brag too much (Moss-Racusin and Rudman, 2010).

## Good girls are modest

The societal adage “Good girls don't brag,” aligns with gender stereotypes linking manhood with personal agency encouraging men to emphasize their successes, whereas femininity is stereotypically linked with communality, encouraging women to stay modest (Moss-Racusin and Rudman, 2010). If adhering to cultural stereotypes, where women are expected to be acquiescent and modest, results in social acceptance, women may be unmotivated to self-promote in an effort to seem humble. The backlash avoidance model (BAM) (Moss-Racusin and Rudman, 2010) suggests women insufficiently self-promote because they fear backlash for such behavior that is incongruent with their traditional gender roles (Eagly and Karau, 2002). Not being gender congruent can lead to interpersonal and economic sanctions against “deviants,” as women who self-promote and brag about their successes are seen as not fitting the gender prescriptions for women. As a result gender atypical women are not liked and are seen undeserving promotion or a raise (Rudman, 1998; Heilman et al., 2004; Rudman and Fairchild, 2004; Moss-Racusin and Rudman, 2010; Rudman and Phelan, 2010). Self-promotion is key to not only finding jobs, but well-paying jobs (Rudman, 1998; Eagly and Karau, 2002; Heilman and Okimoto, 2007), but women who actively promote themselves are perceived as equally competent as men, but with less social skills. Nevertheless, only among female candidates (not male ones) perceived social skills are potentially a stronger predictor for hiring decisions than perceived competency (Phelan et al., 2008).

Women seem to be aware of the potentially negative consequences of counter-stereotypical gender behavior and they demonstrate more modest self-presentation strategies than men (Carli, 1990; Tannen, 1994). For fear of being perceived as too demanding women use strategies that allow them to avoid directly detailing why they deserve a higher salary and they seem to accept the first salary offer they hear during a job interview and they (Stuhlmacher and Walters, 1999; Babcock and Laschever, 2003). Also studies show that managers prefer working with amicable women, who do not negotiate offers during the interview processes, but accept that behavior among men (Bowles et al., 2007). It thus seems that women's tendency to adopt more modest presentation skills is more demanded by managers.

Nevertheless, these are career-climbers that get promoted because they often emphasize their strengths (Babcock and Laschever, 2003). Summing up, while self-promotion is essential for women to overcome negative stereotypes about possible perceived incompetence and lack of leadership skills, they must also navigate the double standard that self-promoting behavior is more acceptable for men than for women (Moss-Racusin and Rudman, 2010). As a result women may feel they must choose between a successful career, and social acceptance (Heilman, 2001; Phelan et al., 2008; Moss-Racusin and Rudman, 2010).

## Gendered self-descriptions are changing

Agency is dimension of social perception stereotypically related to being successful and associated with being a man (Vandello and Bosson, 2013). Its core entails efficacy and effectiveness and is demanded from men and not from women (Prentice and Carranza, 2002; Kosakowska-Berezecka, 2012; Rudman et al., 2012). However, recent meta-analyses of self-descriptions of men and women in U.S. across two time frames (Donnelly and Twenge, 2017) show that women's agency ratings increased significantly between 1993 and 2012, whereas their communion ratings remained relatively stable. No significant changes were observed for men. This trend of women's increase in endorsement of agency in their self-descriptions is anticipated to continue (Twenge, 1997; Diekman and Eagly, 2000; Diekman et al., 2005; Morton et al., 2011). Hence self-promotion may not be as unacceptable for women as research previously indicated. Nevertheless, several studies indicate that personal agency in women should be accompanied with communality as women are evaluated more favorably when they display a mix of agentic and communal qualities (Carli et al., 1995; Carli, 2001). For example, self-promoting, yet non-domineering, women were not at risk for backlash (Rudman and Glick, 2001). Nevertheless, while women may need to be both agentic and communal to avoid backlash, solely agentic men are rewarded both socially and economically (Phelan et al., 2008; Moss-Racusin and Rudman, 2010) hence men's tendency to self-promote is more expected and more demanded from them.

## Self-promotion, fear of backlash and anticipation of success

Being not so stereotypically inclined to self-promote women might also underestimate their chances to succeed and reach high performance. Sharon Sandberg, chief operating officer at Facebook from 2008, in her presentation for TED.com in January 2010 mentions a story about her and her female friend who felt dissatisfied with their performance in a test, whereas her brother, who actually spent less time on preparation was 100% positive that he will receive the highest score in the class (Sandberg, 2010). Several studies conducted in the 80s indicate that women underestimate their skills and opportunities to succeed more than men do (Gold et al., 1980; Erkut, 1983) and this is the result of their self-stereotyping (Ehrlinger and Dunning, 2003).

As both wealth and success are typically linked with masculinity, women might also potentially fear the negative consequences of agency, dominance and success. In the 1970's, Matina Horner asked men and women to finish the story about a character, a male or female student, who scored well on a test in a typically masculine or feminine field. Her results showed that women, more often than men, finished the story in a pessimistic way, anticipating the female character would not be socially accepted. This is connected to the fear of success (FOS) (Horner, 1972; Cherry and Deaux, 1978), which manifests itself in anticipating negative consequences of success due to gender incongruence, such as losing femininity, self-esteem and social acceptance (Horner, 1972). FOS results from cultural stereotypes or beliefs (Feather and Raphelson, 1974; Hyland et al., 1985) and this fear of a backlash for gender incongruent behavior such as, e.g., self-promotion is due to an awareness of proscribed behavior for women. FOS may be related to less effective self-promotion techniques and lower expectations of success among women and thus could also influence women to take lower paying jobs (Major et al., 1984a; Major and Forcey, 1985; Major and Testa, 1989).

People evaluate their own and other persons' abilities all the time (Freund and Kasten, 2012). There also exist a well-documented effect of gender differences with regard to the level of self-estimates of cognitive abilities, usually suggesting that women tend to underestimate and men tend to overestimate their own levels of ability (Szymanowicz and Furnham, 2011). This effect is often associated with gender stereotype threat—activation of a negative stereotype for members of a group (in our case women, not being able enough) may both (1) impede their actual test performance but also (2) lead to a bias into the self-estimation process and inaccurate and unstable assessment of one's cognitive abilities (Steele, 1997; Aronson and Inzlicht, 2004).

On the other hand meta-analysis performed by Freund and Kasten (2012) seems to indicate that women actually do not differ from men in self-appraisals concerning their skills (Freund and Kasten, 2012). Our research thus aims to verify whether women's lower expectations concerning their future earnings and chances for success may be the result of adopted modest self-presentation strategies and fearing negative consequences of success. As gender stereotypes strongly link wealth with men (Williams et al., 2010), similarly success is linked to men and thus the field of success might not be congruent with women's social roles and as such might be potentially avoided by them.

Past studies have put more emphasis on women as being more predisposed socially to experience fear of success (Feather and Raphelson, 1974; Zuckerman and Allison, 1976; Pedersen and Conlin, 1987; Fried-Buchalter, 1997). However, Cherry and Deaux (1978) suggest that associating success with negative consequences mainly reflects a reasonable fear of the consequences of violating gender-appropriate ways, and can be seen in both women and men—women fear success more and are more inclined to avoid it (see also (Hyde, 1996)). On the other hand psychologists of women have concluded that there is no solid research evidence to support the idea that women are any more or less driven toward success than men, given similar opportunities for advancement (Yoder, 1999), so in our study we also aim to analyze gender differences with regard to levels of fear of success.

Our work expands upon previous studies by analysing how men and women anticipate their own future salaries and success in performance (Studies 1 and 2) and we relate it to both women's and men's anticipation of negative consequences of success and inclination to self-promote. We analyzed whether lower propensity to self-promote and fear of success were related to lower expectations for future earnings and academic performance for two demographics—college students and high school pupils. We expected men and women would differ in their inclinations to manifest self-promotion strategies and that they will differ with regard to the extent they associate success with negative consequences—women would be less willing to self-promote and would fear success more than men. We also tested the hypothesis that fear of success and self-promotion will be predictors of lower salaries and anticipation of lower chances of success in an exam.

Study 1 focused on Polish students of management and economics. Graduates of these two prominent fields are expected to have high salaries and reach high positions in their careers. The number of female students studying both management and economics is rising every year and in 2016 women constituted over 60% of students in economics departments (Concise Statistical Yearbook of Poland, 2016), nevertheless these are male graduates who earn more than female graduates and reach higher positions in their careers (Kijewska, 2017). We recorded the students' predictions concerning their academic success and their future earning potential and we measured our participants' levels of tendency for self-promotion and fear of success. In Study 2, we conducted our study among high school students to verify whether associating success with negative consequences and differences in self-promotion strategies manifests itself in a younger demographic. As there is a considerable gap in gender studies with non-American population samples we also wanted to test our assumptions in a European context, which adds value to presented line of research.

We suspected that in both studies 1 and 2 women will demonstrate lower expectations concerning their future earnings than men (hypothesis 1a) and will anticipate lower chances for success in an exam (hypothesis 1b) in comparison to men. As research indicates that women do not differ from men in their self-appraisals concerning their abilities (Freund and Kasten, 2012) but following backlash avoidance model (Moss-Racusin and Rudman, 2010) we expect that men and women will differ with regard to self-presentation strategies and that women will tend to be more modest than men and less likely to self-promote (hypothesis 2) (Moss-Racusin and Rudman, 2010) and that women will associate success with more negative consequences and will rate their fear of success as higher than men (hypothesis 3) (Feather and Raphelson, 1974; Zuckerman and Allison, 1976; Pedersen and Conlin, 1987; Fried-Buchalter, 1997). We also suspected that the adopted self-presentation strategies will be predictors of both anticipation of one's earnings (hypothesis 4a) and success in exam performance (hypothesis 4b). We also predicted that the fear of success would predict both participants anticipated future salary (hypothesis 5a) and their anticipated success in test performance (hypothesis 5b).

As contemporary women, in comparison to 1970's are more likely to endorse agentic traits in their self-descriptions (Donnelly and Twenge, 2017) hence we might assume that differences between women and men with regard to inclination to self-promote and fear of success might be less visible among younger groups than older groups (hypothesis 6). In order to verify this hypothesis we conducted our studies in two age groups – students (study 1) and high school pupils (study 2).

## Study 1

### Method

#### Participants

We analyzed the results of the questionnaire collected from 234 undergraduate students (108 men and 126 women, age: *M* = 22.32, *SD* = 2.23) recruited from the Faculty of Management and Faculty of Economics at a prominent Polish university.

#### Procedure

This study was carried out in accordance with the recommendations of Institutional Review Board of the Institute of Psychology, University of Gdansk ethical committee. Prior to taking the exam, students were asked to participate in the study on a voluntary basis and were informed that their participation was anonymous and had no influence on their future exam results. The questionnaire included an information sheet that informed the participants that their completion of the questionnaire will be understood as consent for the use and publication of the data.

#### Materials

To measure the variables, we used a questionnaire composed of the following scales:

##### Anticipated exam results

Participants were asked to predict their exam results on a scale from 1 to 6 (such as *What will be the percentage of correct answers you'll have in the exam you are about to take?*), where 1 meant “less than 50%” and 6 meant “from 90 to 100%.”

##### Anticipated future earnings

Participants were asked to predict the level of their monthly salaries in three time periods: (1) right after graduation, (2) 5 years after graduation, and (3) 10 years after graduation. While answering questions regarding these three time periods, the participants used a 6-point scale indicating the level of their monthly salaries (1 = less than 1,500 PLN, approximately 500 U.S. dollars (USD); 6 = more than 5,500 PLN, approximately 1,800 USD).

##### Fear of success

To measure the extent to which men and women associated success with negative consequences Polish translation of the 27-item Fear of Success Scale (FOSS), developed by Zuckerman and Allison (1976), assessed individual differences in motivation for avoiding success. Answers were given on a 7-point Likert scale from 1 (*strongly disagree*) to 7 (*strongly agree*). The FOSS contained items measuring the benefits of success (such as *Achievement commands respect*), the presumed costs of success (such as *Often the cost of success is greater than the reward*) and the relative value of success in comparison to their alternatives (such as *The rewards of a successful competition are greater than those received from cooperation*). By generating a single score, Zuckerman and Allison implicitly assumed all FOSS items represented a single fear of success construct, but so far the factor analysis results do not support this assumption (Metzler and Conroy, 2004). Using exploratory factor analyses, Fried-Buchalter (1992) found nine factors with eigenvalues greater than 1.00 but interpreted a three-dimensional model of fear of success scores comprising: (a) negative impacts on self-evaluation and affect (b) external costs of success, and (c) positive perceptions of competition. The first two components of this multidimensional model of FOSS scores converged with recent theoretical developments (Metzler and Conroy, 2004). Using data from our studies we conducted a series of confirmatory factor analyses (CFA) using WLSMV estimator (for ordinal data) to test models of FOSS scores from the literature. We specified a one-factor model by loading all items onto one latent factor. We also specified three-factor model mentioned above. Finally, we decided to accept a one-factor model consisting of 13 items from two subscale (negative impacts on self-evaluation and affect and external costs of success) found by Fried-Buchalter (1992) [see Table A1, Appendix A (Supplementary Material)] with the best-fit measures: χ^2^(*df*) = 136.55 (65), CFI = 0.90, RMSEA (90% C.I) = 0.058 (0.044–0.071), SRMR = 0.054. The reliability of 13-items FOSS in the Study 1 was 0.79. Since the data is ordinal we used the factor score of variable to enable the use of parametric tests in subsequent analyses.

##### Self-presentation: self-promotion and self-depreciation

The Self-presentation Style Questionnaire (SSQ), developed by Wojciszke (2002), assessed the individual's tendency to self-promote and self-depreciate. The original questionnaire consisted of 30 items (15 for each of the two subscales, e.g., *I emphasize my abilities* (self-promotion); *I underestimate the importance of my achievements* (self-depreciation). Answers were given on a 5-point scale from 1 (*never*) to 5 (*very often*). We used the factor score of variable in subsequent analyses. Using data from our both studies we conducted a confirmatory factor analyses (CFA) using WLSMV estimator to test two-factor model of self-presentation [see Table A2, Appendix A (Supplementary Material)]. From both scales we removed items with the lowest factor loadings. The final 17-item model was well fitted to the data: χ^2^(*df*) = 186.69 (117), CFI = 0.91, RMSEA (90% C.I) = 0.043 (0.031–0.055), SRMR = 0.060. The reliability of self-promotion and self-depreciation scales in the Study 1 was 0.76 and 0.73 respectively.

### Data analysis

In Study 1, single questions with ordinal response scale were used to measure anticipated exam results and anticipated future earnings in three time perspectives. Therefore, non-parametric test such as the rank-based Mann-Whitney-Wilcoxon was used to compare the way men and women estimate their chances for success in the exam and how much they will earn in the future (hypotheses 1a and 1b). For testing gender differences in fear of success, self-promotion and self-depreciation (hypotheses 2 and 3) we used the factor scores to enable the use of parametric procedure (*t*-test). Finally, we used ordinal logistic regression to predict the anticipated exam results and the anticipated future earnings in three time perspectives (ordinal dependent variables) given gender, fear of success, and self-promotion as predictors (hypotheses 4 and 5). In order to capture the ordered nature of dependent variables we based on cumulative odds ordinal logistic regression with proportional odds, which uses cumulative categories. For calculations, we used the R environment (R Core Team, 2017) with the lavaan package (Rosseel, 2012) as well as the IBM SPSS Statistics 24 software.

### Results

Tables 1, 2 displays the distribution of answers to questions about anticipated exam results and anticipated earnings it three time perspectives in a group of male and female. The results of Mann-Whitney-Wilcoxon test showed that the distribution of anticipated exam results as well as distributions of anticipated earnings in three time periods were significantly different among men and women. As expected, female students estimated their future exam results and earnings at lower levels compared to their male counterparts.

**Table 1:** Distribution of answers to questions about anticipated exam results in the Study 1.

**Anticipated exam results**	**Male^a^**	**Female^b^**	**Total^c^**
	**%**	**%**	**%**
1/less than 50%	4.6	11.9	8.5
2/50–60%	20.4	34.1	27.8
3/60–70%	17.6	16.7	17.1
4/70–80%	21.3	15.9	18.4
5/80–90%	16.7	8.7	12.4
6/90–100%	19.4	12.7	15.8
Median	4.0	3.0	3.0

a N = 108;

b N = 126;

c *N = 234*.

**Table 2:** Distribution of answers to questions about anticipated earnings in the Study 1.

	**Right after graduation**			**Five years after graduation**			**Ten years after graduation**		
	**Male**	**Female**	**Total**	**Male**	**Female**	**Total**	**Male**	**Female**	**Total**
**Anticipated earnings**	**%**	**%**	**%**	**%**	**%**	**%**	**%**	**%**	**%**
1/ less than 1,500 PLN	6.5	15.2	11.2	0.0	0.0	0.0	0.0	0.0	0.0
2/1,500–2,500 PLN	38.9	48.8	44.2	8.4	19.2	14.2	5.7	7.3	6.5
3/2,500–3,500 PLN	27.8	27.2	27.5	28.0	36.8	32.8	10.4	29.8	20.9
4/3,500–4,500 PLN	12.0	6.4	9.0	18.7	24.8	22.0	11.3	19.4	15.7
5/4,500–5,500 PLN	0.9	0.8	0.9	16.8	12.0	14.2	19.8	16.9	18.3
6/more than 5,500 PLN	13.9	1.6	7.3	28.0	7.2	16.8	52.8	26.6	38.7
Median	3.0	2.0	2.0	4.0	3.0	4.0	6.0	4.0	5.0

*N _Male_ = 108; N _Female_ = 126; N _Total_ = 234*.

As shown in Table 3, women showed a greater fear of success than men. However, there were no significant differences in the self-promotion and self-depreciation strategies between men and women. Additionally, the correlation analysis showed that the higher fear of success than higher self-depreciation (*r* = 0.30, *p* < 0.01). There was no significant correlation between fear of success and self-promotion.

**Table 3:** Gender differences in fear of success, self-promotion and self-depreciation in the Study 1.

	**Male^a^**		**Female^b^**		
**Variable**	***M***	***SD***	***M***	***SD***	***t***
Fear of success	−0.02	0.56	0.17	0.53	−2.61^**^
Self-promotion	0.06	0.49	0.06	0.59	−0.04
Self-depreciation	−0.01	0.34	0.00	0.36	−0.39

a *N = 108*.

b *N = 126*.

** *p < 0.01. We used the factor score of all variables*.

Table 4 contains the estimated coefficients for the predictor variables in tested models. For all four dependent variables, the coefficient for gender (coded −1 = male, 1 = female), the independent variable in the model, for the value of −1 is positive. That means women anticipated poorer performance on the exam and lower future earnings compared to men. As we predicted self-promotion was significant predictor of both anticipated exam results and future earnings. Regardless of gender, higher self-promotion is associated with expected higher success. It is worth noting that self-promotion rather than self-depreciation is a significant predictor of predicting future successes (in the models that we tested the relationships between self-depreciation and other dependent variables were not statistically significant). The fear of success was a significant predictor only for anticipated earnings right after graduation. After removing the other two variables (gender and self-promotion) from the models, the fear of success actually predicted anticipated earnings.

**Table 4:** Results of ordinal logistic regression analyses for the anticipated exam results and earnings in three time periods in the Study 1.

**Dependent variable**	**Predictors**	**Estimate**	**S.E**.	**Wald**	***df***	**Sig**.
Anticipated exam results (Model 1)	Fear of success	−0.16	0.22	0.53	1	0.47
	Self-promotion	0.57	0.22	6.61	1	0.01
	Gender = −1 (male)	0.72	0.25	8.52	1	0.00
Anticipated earnings right after graduation (Model 2)	Fear of success	−0.51	0.23	4.83	1	0.03
	Self-promotion	0.55	0.23	5.50	1	0.02
	Gender = −1 (male)	0.89	0.26	11.84	1	0.00
Anticipated earnings 5 years after graduation (Model 3)	Fear of success	−0.20	0.22	0.78	1	0.38
	Self-promotion	0.90	0.23	15.23	1	0.00
	Gender = −1 (male)	1.02	0.26	15.98	1	0.00
Anticipated earnings 10 years after graduation (Model 4)	Fear of success	−0.30	0.23	1.68	1	0.19
	Self-promotion	74	0.23	10.14	1	0.00
	Gender = −1 (male)	1.13	0.26	18.53	1	0.00

*Model 1: Goodness of fit χ^2^(df) = 1,114.38 (1,107), p = 0.43, Cox and Snell Pseudo R^2^ = 0.07; Model 2: Goodness of fit χ^2^(df) = 1,096.31 (1,102), p = 0.54, Cox and Snell Pseudo R^2^ = 0.10; Model 3: Goodness of fit χ^2^(df) = 869.39 (877), p = 0.57, Cox and Snell Pseudo R^2^ = 0.15; Model 4: Goodness of fit χ^2^(df) = 895.56 (869), p = 0.26, Cox and Snell Pseudo R^2^ = 0.15*.

### Discussion

Study 1 demonstrated that female students estimated both their future exam results and earnings at lower levels compared to their male counterparts (thus confirming hypothesis 1a and 1b). At the same time, their fear of success in general was greater than for men, confirming hypothesis 3. However, we observed no differences in self-promotion strategies between men and women, which indicates that our female participants were not more modest than our male participants with regard to their self-promoting strategies. Nevertheless, our results also showed that self-promotion is predictor of both anticipated exam results and future earnings for both female and male students of economics and management.

As self-promotion can be linked to one's ratings of agency (Prentice and Carranza, 2002; Kosakowska-Berezecka, 2012; Rudman et al., 2012), we wanted to verify whether individuals self-stereotyped by prioritizing communality or agency in Study 2 (Phelan et al., 2008; Moss-Racusin and Rudman, 2010). These prioritizations might also be potential predictors of both anticipation of future earnings and exam results. Several studies indicate that differences between women and men in their self-descriptions are decreasing especially with regard to their self-rated agency, hence the way they describe themselves might be different for younger generations (Donnelly and Twenge, 2017). Therefore, Study 2 was conducted among high school pupils to verify whether (1) at this point of life women and men already manifest differences with regard to anticipation concerning future earnings and academic success and (2) their anticipation of future earnings and academic success will be predicted by their self-promotion strategies, agency vs. communality self-descriptions and fear of success.

## Study 2

In Study 2, we sought to establish whether self-promotion strategies, along with agentic and communal self-stereotyping and fear of success were related to anticipating future earnings and academic success. As Study 2 was conducted with younger students, we wanted to verify whether similar relationships as in Study 1 will be observed among younger pupils and that FOS, self-promotion strategies, along with self-ratings of agency and communality, would predict a school pupils' anticipation of academic and financial success and whether this would be moderated by their gender (see hypotheses 1-5 from study 1). We also tested hypothesis 6, in which we wanted to see whether self-stereotyping (agency and communality) will be also predictors of anticipated future earnings and academic success. In this study we have analyzed pupils perceived chances for success in two fields: stereotypically female congruent field of language and stereotypically male congruent field of mathematics (Halpern et al., 2007)—both courses are obligatory in Polish high school and in order to graduate from high schools pupils are taking mathematics and language exams. The results from these exams later determine their university options.

### Method

#### Participants

We analyzed the results of the questionnaire collected from 100 high school pupils (40 men and 60 women, age from 18 to 19 years old) recruited from one of the schools in Pomerania, Poland—the graduates of this school are also potential students of economics and management, the group where study 1 was conducted.

#### Procedure

This study was carried out in accordance with the recommendations of Institutional Review Board of the Institute of Psychology, University of Gdansk ethical committee. Prior to taking the exam, students were asked to participate in the study on a voluntary basis and were informed that their participation was anonymous and had no influence on their future exam results. The questionnaire included an information sheet that informed the participants that their completion of the questionnaire will be understood as consent for the use and publication of the data.

The data were collected 3 weeks before their end-of-year final exams. As mentioned before these results would determine their university options. All pupils were asked to participate in the study on a voluntary basis and complete the questionnaire. They were informed that their participation was anonymous and their answers in the questionnaires provided will have no influence on their future exam results. The questionnaire also included an information sheet that informed the participants that their completion of the questionnaire would be understood as consent for the use and publication of the data. The school authorities also approved the content of the questionnaire.

#### Materials

To measure the variables, we used a questionnaire composed of the following scales:

##### Anticipated exam results

Participants were asked to predict, on a percentage scale, the results of their final high school exam for two obligatory subjects: language and mathematics.

##### Anticipated earnings

Participants were asked to predict their monthly salaries (in PLN) in three time periods: right after graduation, 5 years after graduation and 10 years after graduation.

##### Self-stereotyping scale

To measure personal self-stereotyping regarding agency and communality we employed a Polish version of a 13-item agentic (7 items, e.g., *I am assertive, competent, self-confident*) and communal (6 items, e.g., *I am warm, caring, sensitive*) scale based on measurements by Laurin et al. (2011). The participants were asked to decide using a 1–9 Likert type scale the extent to which they agree that a given trait describes them (from 1, *strongly disagree* to 9, *strongly agree*). The scale was translated into Polish using a back-translation process. Since the data is ordinal we used the factor score of variable to enable the use of parametric tests in subsequent analyses. Using data from Study 2 we conducted a confirmatory factor analyses (CFA) using WLSMV estimator to test two-factor model of self-stereotyping [see Table A3, Appendix A (Supplementary Material)]. The original model was well fitted to the data: χ^2^(*df*) = 78.92 (64), CFI = 0.92, RMSEA (90% C.I) = 0.049 (0.001–0.081), SRMR = 0.083. The reliability of agency and communality scales in the Study 2 was 0.83 and 0.79 respectively.

To measure fear of success and self-presentation strategies we used the same measures as in Study 1: FOSS and SSQ. The reliability of FOSS in the Study 2 was 0.81 and the reliability of self-promotion and self-depreciation scales in the Study 2 was 0.79 and 0.70 respectively.

### Data analysis

Compared to the Study 1, in the Study 2 we used continuous single-item scales to measure all dependent variables: anticipated exam results and anticipated future earnings in three time perspectives. Therefore, parametric test (*t*-test) was used to compare the way men and women estimate their chances for success in the exam and how much they will earn in the future. For testing gender differences in fear of success, self-presentation (self-promotion and self-depreciation) and self-stereotyping (agentic and communal) we used the factor scores of the variables to enable the use of parametric procedure (*t*-test). Finally, we used linear regression to predict the anticipated exam results and the anticipated future earnings in three time perspectives (dependent variables) given gender, fear of success, and agentic and communal as predictors.

### Results

Table 5 display means, standard deviations, and correlations of all dependent variables. As we can see, the average expected result of the language test is lower than the math test. The average salary expected by the students right after graduation (*M* = 2,724.76; approximately 850 USD) was more than three times lowers than those expected 10 years after graduation (*M* = 8,792.22 PLN; approximately 2,750 USD). It is worth noting that long-term expectations far outweigh the average remuneration in Poland (4,353.55 PLN; Central Statistical Office of Poland, 2016). The anticipated result on the exams does not correlate or low correlate with the anticipation of future earnings. Both measures can therefore be regarded as independent indicators of success.

**Table 5:** Descriptive statistics and intercorrelations of dependent variables in the Study 2.

**Variable**	***N***	***M***	***SD***	**1**	**2**	**3**	**4**
1. Anticipated result on the exam—language	100	69.45	12.13	–			
2. Anticipated result on the exam—math	100	83.14	13.61	0.06	–		
3. Anticipated earnings right after graduation	87	2,724.76	1,551.27	−0.04	0.26^*^	–	
4. Anticipated earnings 5 years after graduation	90	5,093.33	3,726.21	0.17	0.12	0.52^**^	–
5. Anticipated earnings 10 years after graduation	90	8,792.22	6,137.91	0.16	0.17	0.51^**^	0.83^**^

* p < 0.05;

** *p < 0.01*.

Table 6 present gender differences in all tested variables in the Study 2 (results of the *t*-test for independent sample). As expected, younger women estimated their future earnings at lower levels than younger men, but only for periods immediately after school and 5 years after graduation. In the 10 years perspective, there were no differences between women and men in their levels of anticipated earnings. While there were no gender differences for the expected result in the final math exam, high school girls expected higher scores in their language exams than high school boys. The study also found no significant gender differences regarding self-promotion, self-depreciation, and fear of success or agency self-ratings. At the same time, high school girls rated their communality higher than high school boys and showed stronger inclination for.

**Table 6:** Gender differences in measured variables in Study 2.

**Variable**	**Male^a^**		**Female^b^**		
	***M***	***SD***	***M***	***SD***	***t***
Anticipated result on the exam—language	64.53	12.58	72.73	10.72	−3.50^**^
Anticipated result on the exam—math	81.33	14.66	84.35	12.85	−1.09
Anticipated earnings after graduation	3,175.67	1,669.20	2,406.47	1,392.47	2.34^*^
Anticipated earnings 5 years after graduation	6,143.24	4,941.47	4,360.38	2,351.05	2.29^*^
Anticipated earnings 10 years after graduation	9,816.22	6,391.55	8,077.36	5,910.28	1.33
Fear of success^c^	−0.22	0.61	−0.17	0.54	0.45
Self-promotion^c^	−0.01	0.56	−0.22	0.57	1.80
Self-depreciation^c^	−0.03	0.29	0.03	0.43	−0.72
Agency^c^	−0.07	1.08	0.05	0.93	−0.60
Communality^c^	0.32	0.91	0.21	0.75	−3.17^**^

a *N = 40*.

b *N = 60*.

* p < 0.05;

** *p < 0.01*.

c *Factor score of variables*.

The correlation analysis confirmed that the higher fear of success than higher self-depreciation (*r* = 0.38, *p* < 0.01) and there was no significant correlation between fear of success and self-promotion, agency and communality. Agency self-rating positively correlated with self-promotion (*r* = 0.42, *p* < 0.01) and communality self-rating (*r* = 0.48, *p* < 0.01) and negatively with self-depreciation (*r* = −0.25, *p* < 0.05). There was no significant relationship between communality and both self-presentation strategies.

In the group of high school students, we found that fear of success and agency were predictors of anticipated future earnings right after graduation and 5 years after graduation. The results of the hierarchical regression analysis are presented in Tables 7, 8.

**Table 7:** Results of hierarchical regression analyses for the anticipated future earnings right after graduation.

**Model**	**Predictors**	***B***	***S.E*.**	***Beta***	***t***
1	(Constant)	2, 688.89	164.59		16.34^**^
	Gender	−337.19	157.12	−0.22	−2.15^*^
	Fear of success	−591.44	268.90	−0.22	−2.20^*^
	Agency	424.59	161.34	0.27	2.63^**^
2	(Constant)	2, 685.19	161.83		16.59^**^
	Gender	−358.80	154.86	−0.23	−2.32^*^
	Fear of success	−627.60	265.01	−0.24	−2.37^*^
	Agency	419.12	158.65	0.26	2.64^**^
	Gender ^*^ Agency	−311.41	159.03	−0.20	−1.96^*^

* p < 0.05;

** *p < 0.01; Model 1 fit: F_(3, 86)_ = 5.66 (p < 0.01); R = 0.41; Adjusted R^2^ = 0.14; Model 2 fit: F_(4, 86)_ = 5.35 (p < 0.01); R = 0.46; Adjusted R^2^ = 0.17 (ΔR^2^ = 0.04; p < 0.05)*.

**Table 8:** Results of hierarchical regression analyses for the anticipated future earnings 5 years after graduation.

**Model**	**Predictors**	***B***	***SE***	**Beta**	***t***
1	(Constant)	5, 153.68	372.57		13.83^*^
	Gender	−904.19	355.99	−0.24	−2.54^*^
	Fear of success	−1660.70	612.44	−0.26	−2.71^**^
	Agency	1, 332.51	368.67	0.34	3.61^**^
2	(Constant)	5, 146.66	364.45		14.12^**^
	Gender	−973.17	349.63	−0.26	−2.78^**^
	Fear of success	−1762.77	600.87	−0.27	−2.93^**^
	Agency	1, 314.83	360.72	0.34	3.65^**^
	Gender ^*^ Agency	−795.53	361.78	−0.20	−2.20^*^

* p < 0.05;

** *p < 0.01; Model 1 fit: F_(3, 86)_ = 9.32 (p < 0.01); R = 0.49; Adjusted R^2^ = 0.22; Model 2 fit: F_(4, 86)_ = 8.51 (p < 0.01); R = 0.54; Adjusted R^2^ = 0.26 (ΔR^2^ = 0.04; p < 0.05)*.

As Tables 7 shows, fear of success and agency were significant predictors of anticipated earnings just after graduation (Model 1). Additionally our results show an interaction effect between gender and agency in predicting future earnings (Model 2). Regression analysis performed separately in the group of women and men showed that the agency (taking into account FOS in the model) is a significant anticipation of future earnings but only for young men (β = 0.47, *p* < 0.01). In the group of women there was no significant relationship between the agency and the predictions concerning future earnings (β = 0.06, *p* = 0.64). Very similar results were obtained for anticipated earnings 5 years after graduation: β = 0.47 and 0.20 respectively. The results of our study have shown that neither self-promotion nor self-depreciation are significant predictors of expected success (note that self-depreciation strongly correlates with FOS, which may indicate a common variance in predicting the dependent variable).

### Discussion

Study 2 results show that high school boys and girls anticipate to obtain similar results with regard to their math exam results. We have also showed that school girls view their chances for success in the language exam as higher than school boys, thus following gender congruent expectations concerning girls higher language skills (Halpern et al., 2007). They also seem to differ regarding their future earnings but only in the short term. For the 10-year marker, there were no observable differences between anticipated earnings for girls and boys. Our results also show that girls and boys do not differ with regard their expectations that success will bring negative consequences and have similar self-ratings within self-promotion and agency, but girls rate themselves as more communal than men. As for anticipating future earnings, only fear of success (strongly correlating with self-depreciation) and agency, but only for young men, were significant predictors of earnings, but only after graduation. Neither self-promotion nor self-depreciation were significant predictors of expected success in an exam.

Our results might find a potential explanation in the fact that school pupils have not yet experienced what is it like to be in the labor market and their perceptions concerning their situation in the labor market might be too abstract in comparison to students. Another study analyzed the perception of gender pay gap in high schools, and it showed that high school pupils actually do not consider gender pay gap as their problem (Kosakowska-Berezecka et al., forthcoming). Additionally these results could be analyzed in the context of generational gap comparisons—younger generations of Polish women might feel more entitled to be successful than older ones, on equal levels of men. Analysing Polish culture through the lens of ongoing transit of values from more collectivistic values of modesty, it might be visible that young people in Poland are being discouraged from direct self-promotion and instead encouraged to endorse more individualistic attitude to life and career. Younger generations, as opposed to the older ones, are more expected to manifest their skills and talents (Spector et al., 2001; Zawadzka et al., 2016).

## General discussion

The most visible gender pay gap in the labor market is observed among women and men occupying managerial positons—women earn 68% of what men earn at these positions in Poland (Kijewska, 2017). When it comes to lower positions in companies women earn 92% of what men earn. Women seem to be aware of this existing gender pay gap. In the 1980s, with a sample of 50 management students, Major and Konar (1984) found that when pre-career women and men predicted the level of pay they expected to receive upon entering their careers and at career peak, the expectations of women were lower than those of men for both time periods. It was found that the early - career pay expectations of women were only 83.5% of what men expected to be paid, and women's peak-career pay expectations were only 54% of men's. More than 30 years later women's predictions haven't changed—they still predict they will earn less than men as our study results imply, especially when looking at the results obtained among students sample of economy and management. Economy and management female students expected to earn less and achieve less in an exam than male students, and these results were predicted by their declared levels of self-promotion and fear of success, with the latter rated as higher by women than by men.

Women's pay expectations are potentially lower than those of men because women and men have different social comparison standards, have different perceptions regarding the value of their work effort (Major et al., 1984a). Predictions concerning one's future financial success that originate in negative stereotypes about oneself are accompanied by the awareness of the gender pay gap and segregation of the labor market with respect to sex. This in turn might maintain women's position as underpaid workers (Walton and Cohen, 2007; Walton and Spencer, 2009). If we also take into consideration the fact that the fear of being perceived as too demanding and dominant is an accurate explanation for differences in the efficiency of negotiations carried out by men and women (Amanatullah and Morris, 2010) focusing not only on women's self-promotion strategies but also reducing the impact of experienced fear of success are exemplars of themes for potentially effective interventions aiming at empowering women in the labor market (Bowles and Babcock, 2013). Being bold can aid men's competence but hinder women's—one of the goals of educational system should focus on changing this belief among women, as this might increase their anticipated level of success and in turn make the success in their career more tangible. Such educational interventions should take place during individuals educational path—between the high school and university—e.g., one of such activities could be career planning classes for women and men to be familiar with the existing gender stereotypes influencing women's and men's chances for success. Apart from self-promotion, fear of success also functions as a cultural stereotype potentially impeding their chances for success—changing women's beliefs and behavior will not be sufficient for addressing gender inequality in the labor market (in some cases, might have the opposite effect, reducing too demanding and too dominant women's chances for success (Bowles et al., 2007; Phelan et al., 2008). Policy makers should take into consideration the fact that women base their behavior on the existing payment standards, which show women as underpaid workers. What's more women's behavior in which they manifest less self-promotion and demand less is reasonable. If they violate cultural gender norms they experience negative appraisals.

Social status that encompasses success and wealth might be more central to the male than the female gender role (Bosson and Michniewicz, 2013). Gender stereotypes that link men with success, agency and self-promotion and women with communality and being modest are not only descriptive but also prescriptive (Prentice and Carranza, 2002). As a result girls and boys learn that they need to manifest socially accepted gender traits and behaviors (Rudman et al., 2012). Nevertheless, as indicated by more recent research, men's and women's self-descriptions are potentially becoming similar, as women are more likely to endorse agentic traits in their self-descriptions (Donnelly and Twenge, 2017), thus modest self-promotion strategies are also less suitable for women and not that much expected from them. This is also reflected in the results from our study 2 where agency ratings for women and men were not significantly different as well girl's predictions concerning their final language exam scores. The communality scores were higher among schoolgirls but agency was an important predictor for the anticipation of earnings, but only for young men. There might be other potential predictors for anticipation of future success in their earnings and in their performance among school girls and future studies could explore that. Overall high school boys and girls in study 2 expected similar results with regard to their math exam and girls anticipated to have higher scores than boys from their language exams. Both the fact of their lack of professional experience and awareness of situation of women in the labor marker, along with the fact that young people in Poland are more and more expected to manifest their skills and talents (Spector et al., 2001; Zawadzka et al., 2016) might account for the results obtained. On the other hand female students of economics and management do show that FOS and self-promotion play role in their anticipation of future earnings and success, hence being more exposed to labor market, increasing your knowledge about the economic reality of women might increase your tendency to follow gender congruent prescriptions of modest in self-promotion and fear of success. Alternatively (and tentatively), our results might indicate a current generational change that may be occurring, and women may soon ask for more financial compensations than in the past, thus becoming more similar to men with regard to their expected salaries and chances for success.

Our conclusions are limited by the fact that our design did not include the measurement of women's and men's actual performance results. Only with regard to high school pupils we know that overall there were no significant gender differences with regard to their actual math and language exams results. The two groups are not fully comparable as they belong to different groups at different moments of their lives and their awareness of labor market is different. We have also not controlled for levels of test difficulty, importance and perceived gender (in)congruity, hence it would be useful to conduct a similar study but including these three as additional variables. We also have not tested whether women experienced stereotype threat, which could potentially impede their actual performance. Additionally our study was carried out among students of management and economy and pupils of relatively good high school, not yet having full contact with the professional reality of women and more samples varying with their levels of professional experience and different fields of studies should be taken under consideration. Nevertheless, economy and management students too seem to be fully aware of the existing gender stereotypes and they predict their chances for their financial success as lower than their male counterparts and fear success more. Hence, self-promotion won't directly lead to improved career outcomes for women if there's no support from the effective policies, guaranteeing equal pay for both women and men, and not only allowing them equal educational opportunities.

Women's anticipating lower financial success and lower chances for success in an exam is showing that both gender pay gap and lower chances for success are serious and hard-to-solve problem already anticipated by female university students. As they think they will earn and succeed less they will eventually earn less and succeed less (Major et al., 1984b; Small et al., 2007). There is a strong need to give support to women by institutional environment when studying at the university, e.g., by providing trainings for women and men that would protect women from fear of success and lower self-promotion (for examples of interventions promoting gender equality at the university see: de Lemus et al., 2014). But not only institutional support is needed. These are often men who are rather reluctant to accept evidence of gender biases in their organizational environment (Handley et al., 2015) and their role in bringing gender equality cannot be overlooked, especially since they are the ones who hold positions of power. Although majority of gender equality efforts are aimed at women, many studies suggest, that these are men who may inhibit the gender equality in a family or in the organization (Beede et al., 2011; Kosakowska-Berezecka et al., forthcoming). Hence this is important to include men as targets of interventions fostering an emerging culture of gender equality (Holter, 2014). And of the ways to achieve is through showing the benefits of gender equality.

Endorsing less stereotypical approach to women and men in organizations such as universities can clear the air of talent-inhibiting gender stereotypes and allow space for higher performance and innovation.

## Ethics statement

Institutional Review Board approval was obtained from the Institute of Psychology, University of Gdansk ethical committee.

## Author contributions

NK, PJ, TB contributed to the study design. NK and SB collected the data. PJ performed the data analysis and interpretation under the supervision of NK and TB. NK drafted the manuscript, and PJ, TB, and SB provided critical revisions.

### Conflict of interest statement

The authors declare that the research was conducted in the absence of any commercial or financial relationships that could be construed as a potential conflict of interest.
