# Supplementary material for: Self-Presentation Strategies, Fear of Success and Anticipation of Future Success among University and High School Students
Source: Front Psychol. 2017 Oct 27;8:1884. doi: 10.3389/fpsyg.2017.01884 (PMC5663907; doi:10.3389/fpsyg.2017.01884)
Supplement: Supplementary file 1 [file Appendix.docx]

**Appendix A**

Supplemented Materials: Results of the confirmatory factor analysis of used questionnaires

Table A1

*Results of the confirmatory factor analysis of the Success Scale used in both studies*

| **Item** | **Factor loading** |
| --- | --- |
| 2/ Often the cost of success is greater than the reward. [*Zazwyczaj koszt odniesienia sukcesu jest wyższy niż nagroda.*] | .36 |
| 7/ In my attempt to do better than others, I realize I may lose many friends. [*Zdaję sobie sprawę, że próbując być lepszym od innych, mogę stracić wielu przyjaciół*.] | .49 |
| 9/ A person who is at the top faces nothing but a constant struggle to stay there. [*Osoba będąca na szczycie zmaga się z niczym innym, jak z ciągłą walką o pozostanie na nim.*] | .41 |
| 11/ I think ‘success’ has been emphasized too much in our culture. [*Uważam, że „sukces” jest zbyt akcentowany w naszej kulturze.*] | .34 |
| 12/ In order to achieve one must give up the fun things in life. [*Aby coś osiągnąć w życiu, trzeba zrezygnować z przyjemności w życiu.*] | .45 |
| 13/ The cost of success is overwhelming responsibility. [*Sukces wiąże się z przytłaczającą odpowiedzialnością.*] | .53 |
| 16/ A successful person is often considered by others to be both aloof and snobbish. [*Osoba odnosząca sukcesy często jest uważana za powściągliwą i snobistyczną.*] | .57 |
| 18/ People’s behavior change for the worst after they become successful. [*Ludzkie zachowania zmieniają się na gorsze u osób, które odniosły sukces.*] | .63 |
| 19/ When competing against another person, I sometimes feel better if I lose than if I win. [*Kiedy z kimś rywalizuję, czasem lepiej bym się czuł, gdybym przegrał, aniżeli wygrał.*] | .41 |
| 20/ Once you’re on top, everyone is your buddy and no one is your friend. [*Kiedy jesteś na szczycie, wszyscy są twoimi kumplami, ale nikt nie jest przyjacielem.*] | .60 |
| 22/ Even when I do well on a task, I sometimes feel like a phony or a fraud. [*Nawet jeśli dobrze wykonam zadanie, czasem czuję się jak oszust czy naciągacz.*] | .53 |
| 23/ I believe that successful people are often sad and lonely. [*Uważam, że ludzie sukcesu są często smutni i samotni.*] | .60 |
| 25/ When I am on top the responsibility makes me feel uneasy. [*Kiedy jestem na szczycie, odpowiedzialność wywołuje we mnie niepokój*.] | .44 |

*N* = 334.

Table A2

*Results of the confirmatory factor analysis of the Self-Presentation Questionnaire used in both studies*

|  | **Factor loadings** | |
| --- | --- | --- |
| **Item** | ***Self-promotion*** | ***Self-depreciation*** |
| 3/ I emphasize my own merits [Podkreślam własne zasługi.] | .65 |  |
| 4/ I make the impression of being a person occupied with important matters.] [Sprawiam wrażenie osoby zajętej ważnymi sprawami.] | .35 |  |
| 8/ I emphasize my own skills [Podkreślam własne umiejętności.] | .68 |  |
| 11/ I come across as person who knows things [Staram się wypaść na osobę znającą się na rzeczy.] | .51 |  |
| 14/ I easily talk about my successes [Opowiadam o tym, co mi się udało.] | .53 |  |
| 19/ I emphasize my strenghts [Mówię o swoich zaletach.] | .69 |  |
| 27/ When I am not 100% sure about something I don’t show it. [Kiedy brak mi wiedzy na jakiś temat, nie daję tego po sobie poznać.] | .32 |  |
| 28/ I speak decisively, even if I don’t feel fully self-confident [Mówię zdecydowanym tonem, nawet kiedy wcale nie mam pewności.] | .34 |  |
| 30/ When discussing things I like to show that I am knowledgable [W dyskusji lubię pokazać, że się dobrze znam na rzeczy.] | .59 |  |
| 5/ I try to do worse than the other person in order not to hurt her. [Wypadam gorzej od drugiej osoby, żeby jej nie sprawić przykrości.] |  | .43 |
| 12/ I say I am sorry even if this was not really my fault [Mówię przepraszam nawet, kiedy tak naprawdę to nie moja wina.] |  | .42 |
| 13/ I underline my weaknesses [Podkreślam swoje wady.] |  | .64 |
| 17/ When something goes well, I try to show my weaknesses [Gdy coś mi dobrze pójdzie, staram się pokazać także swoje wady.] |  | .55 |
| 18/ I admit I am helpless [Przyznaję się do własnej bezradności.] |  | .53 |
| 22/ I seem to be more helpless than I feel [Zachowuję się bardziej bezradnie niż się czuję.] |  | .37 |
| 25/ In advance I warn others that I won’t be successful with something [Z góry twierdzę, że nie dam sobie z czymś rady.] |  | .47 |
| 29/ When I am successful, I convince others that this was just a coincidence [Kiedy dobrze wypadnę przekonuję innych, że to przypadek.] |  | .52 |
| Covariance: | -.19 | |

*N* = 334.

Table A3

*Results of the confirmatory factor analysis of the Self-Stereotyping Scale used in the Study 2*

|  | **Factor loadings** | |
| --- | --- | --- |
| **Item** | ***Agency*** | ***Communality*** |
| 2/ Assertive [Asertywny] | .54 |  |
| 5/ Intelligent [Inteligentny] | .66 |  |
| 6/ Competent[Kompetentny] | .67 |  |
| 7/ Ambitious [Ambitny] | .57 |  |
| 9/ Competitive [Przebojowy] | .70 |  |
| 10/ Self-confident [Pewny siebie] | .66 |  |
| 12/ Brave [Waleczny] | .68 |  |
| 1/ Kind [Życzliwy] |  | .58 |
| 3/ Emotional [Emocjonalny] |  | .53 |
| 4/ Sensitive[Wrażliwy] |  | .66 |
| 8/ Caring [Troskliwy] |  | .84 |
| 11/ Warm [Ciepły] |  | .68 |
| 13/ Modest [Skromny] |  | .42 |
| Covariance: | .39 | |

*N* = 100.
